# Biosensors for On-Farm Diagnosis of Mastitis

**DOI:** 10.3389/fbioe.2019.00186

**Published:** 2019-07-31

**Authors:** Sofia A. M. Martins, Verónica C. Martins, Filipe A. Cardoso, José Germano, Mónica Rodrigues, Carla Duarte, Ricardo Bexiga, Susana Cardoso, Paulo P. Freitas

**Affiliations:** ^1^Magnomics S.A., Parque Tecnológico de Cantanhede, Cantanhede, Portugal; ^2^INESC Microsistemas e Nanotecnologias Rua Alves Redol, Lisbon, Portugal; ^3^Faculdade de Ciências, CE3C - Centre for Ecology, Evolution and Environmental Changes, Universidade de Lisboa, Lisbon, Portugal; ^4^Faculdade de Medicina Veterinária, Avenida da Universidade Técnica, Lisbon, Portugal; ^5^INL- International Iberian Nanotechnology Laboratory, Braga, Portugal

**Keywords:** dairy industry, mastitis, diagnostics, biosensors, microfluidics, point-of-care

## Abstract

Bovine mastitis is an inflammation of the mammary gland caused by a multitude of pathogens with devastating consequences for the dairy industry. Global annual losses are estimated to be around €30 bn and are caused by significant milk losses, poor milk quality, culling of chronically infected animals, and occasional deaths. Moreover, mastitis management routinely implies the administration of antibiotics to treat and prevent the disease which poses serious risks regarding the emergence of antibiotic resistance. Conventional diagnostic methods based on somatic cell counts (SCC) and plate-culture techniques are accurate in identifying the disease, the respective infectious agents and antibiotic resistant phenotypes. However, pressure exists to develop less lengthy approaches, capable of providing on-site information concerning the infection, and in this way, guide, and hasten the most adequate treatment. Biosensors are analytical tools that convert the presence of biological compounds into an electric signal. Benefitting from high signal-to-noise ratios and fast response times, when properly tuned, they can detect the presence of specific cells and cell markers with high sensitivity. In combination with microfluidics, they provide the means for development of automated and portable diagnostic devices. Still, while biosensors are growing at a fast pace in human diagnostics, applications for the veterinary market, and specifically, for the diagnosis of mastitis remain limited. This review highlights current approaches for mastitis diagnosis and describes the latest outcomes in biosensors and lab-on-chip devices with the potential to become real alternatives to standard practices. Focus is given to those technologies that, in a near future, will enable for an on-farm diagnosis of mastitis.

## Introduction

Mastitis is the inflammation of the mammary gland, most often of infectious origin. It is a painful condition, with serious implications in animal welfare and is one of the most important reasons for cows to prematurely leave the herd. Milk from animals with mastitis cannot be used for human consumption because it has altered chemical composition and organoleptic proprieties (Seegers et al., 2003; Adkins and Middleton, 2018; Ashraf and Imran, 2018). Moreover, milk from infected animals negatively affects the future processing and shelf life of resulting dairy products (Hogeveen et al., 2010a). According to the severity of the inflammation, mastitis can be classified in clinical or subclinical forms. In clinical mastitis, visible manifestations of infection are present, such as abnormal milk (changes in color, presence of clots, flakes), abnormal mammary gland (changes in tissue color, swelling) and changes in animal status (body temperature, appetite, and hydration level). On the other hand, subclinical mastitis is characterized by the absence of detectable clinical signs. Still, milk quality and production yields are usually negatively affected (Adkins and Middleton, 2018; Ashraf and Imran, 2018).

Among the diseases which affect the profitability of production animals, mastitis is considered a major cause of economic loss. Important factors include a decrease in milk production, a decrease in milk quality, discarded milk, premature culling, increased mortality, increased labor, veterinary services, diagnostics, and treatment. Other costs such as risk of other diseases and costs incurring from materials and investments performed for mastitis management should also be considered (Petrovski et al., 2006; Halasa et al., 2007; Hogeveen et al., 2011; van Soest et al., 2016). Several methods have been used to estimate the economic losses associated with mastitis, although differences in modeling approaches, herd/farm variability, world region, and different inputs originated distinct published data. A statistic tool comprising the different mastitis cost factors was developed by Huijps et al. using data from 78 farmers in the Netherlands. Results estimated an average economic loss per mastitis case of €210 but was dependent on the lactation month. Production losses accounted for 71% of the total losses, followed by animal culling (16%), and veterinary activities (1%). Per animal and per year, results varied between €65 and €182. Interestingly, the perception from farmers regarding economic losses was on average €78/cow per year, suggesting that most farmers underestimated the economic burden of mastitis (Huijps et al., 2008). In another study, Bar et al. used a dynamic programing model with data from 5 large herds, in the New York state (600–1,200 milking cows). The animals were followed for 24 months (years 2004–2006). Results pointed to an average cost of a mastitis case at $179 and at $71/animal per year (US currency), with 64.2% of the total cost attributed to milk losses, 7.8% to increased mortality, and 27.9% accounting for treatment costs (Bar et al., 2008). Similarly, Nielsen et al. applied a biodynamic model to a Swedish herd (150 animals), and estimated an average cost per clinical mastitis case of €278 (Nielsen et al., 2010). Recently, Rollin et al. used a deterministic partial budget model to estimate the impact of clinical mastitis within the first 30 days of lactation. Model inputs considered a 1,000 animal herd in the United States of America and a time scale between 2012 and 2014. The average case of clinical mastitis resulted in a total loss of $435 (US currency). Future production losses accounted for 28.7% of total cost; 41,8% attributed to premature culling and animal replacements; 8.3% in treatment costs; 7.4% due to mortality; 5.7% in discarded milk; 4.8% attributed to labor and 3.2% in veterinary services and diagnostics (Rollin et al., 2015). Despite some differences in the overall values, results indicate that mastitis is a disease with a significant and worldwide economic impact.

Notwithstanding the considerable knowledge on etiology and physiology of mastitis, the truth is that it has been proven extremely difficult to control and around 20–30% of dairy cows are diagnosed with a mastitis episode, at least once during lactation (Ganda et al., 2016). Diagnosis of causative agents of mastitis is often not performed, with treatment protocols being applied according to veterinary predefined protocols. The most frequent approach to treatment is the use of systemic or intramammary antibiotic as soon as possible, after detection. Still, the impacts of recurrent antibiotic administration should not be underestimated. The extensive usage of antibiotics and their presence in the environment has received increased concerns due to the raise of antimicrobial resistance-AMR, and consequent adverse effects in human and veterinary health (Aga et al., 2016). Continuous exposure of bacteria to antibiotics may promote selective pressure and genetic exchange of antibiotic-resistance genes leading to prevalence of such resistant populations in the environment (Munita and Arias, 2016). Thus, major concerns deal with the possibility of emerging reservoirs of resistant genes and bacterial populations in food-producing animals, caused by a sustained antibiotic administration. These bacterial populations can then play a key role in the dissemination of resistance genes to other bacterial communities or to bacteria potentially hazardous to humans (Oliver et al., 2010). According to EU data, AMR is responsible for ~25,000 deaths/year and carries an economic burden of €1.5 billion/year in healthcare (https://ec.europa.eu/health/amr/antimicrobial-resistance_en). Mastitis is indeed, the first reason for the use of antimicrobials in dairy cows, with reports of up to 80% of all antimicrobial doses used in dairy cattle being aimed at the treatment or prevention of mastitis cases (Pol and Ruegg, 2010). Oliver et al. published a comprehensive review covering published data regarding antibiotic usage in dairy cows and its impact on antimicrobial resistance in veterinary and human pathogens. They concluded that, although the presence of resistance genes in mastitis pathogens has been documented over the past 40 years, there is no scientific evidence supporting the thesis that the population is progressing (Oliver et al., 2010). On the other hand, during treatment, milk has antibiotic residues that cannot enter the food chain and thus a withdrawal period must be established. In the European Union (EU), the latter is defined considering the maximum residue limit (MRL) for each drug or drug class which is established by the European Medicines Agency (EMA), following a detailed scientific review of the toxicology of each drug or drug class as well as an analysis of the absorption, distribution, metabolism, and elimination of the drug from treated animals. Therefore, the consequences of antimicrobial resistance in dairy pathogens potentially impacting human health are residual as long as safety measurements are applied and milk pasteurized (Oliver et al., 2010). However, several studies, demonstrated an increased prevalence of resistant bacteria in commensal populations from dairy animals undergoing antibiotic treatment (Foutz et al., 2018; Springer et al., 2018). Additionally, a common practice is to use non-salable milk from cows treated with antibiotics to feed young calves which carries the risk of increasing fecal shedding of AMR bacteria. This fact has already been demonstrated in some published works (Brunton et al., 2014; Maynou et al., 2017). Animal manure and runoffs from animal-farm activities are important environmental sources of these compounds and indeed several studies showed a higher prevalence of resistant bacteria, particularly food-borne pathogens (*Lysteria monocytogenes, Salmonella* spp, *Campylobacter jejuni*) and *Enterobacteriaceae* in soil from dairy farms (Oliver et al., 2010). In July 2017, the EU Commission adopted the EU One Health action plan comprising best practices and boosting research in both human/animal health and environmental areas. While further studies are required to confirm the link between antibiotic usage and AMR progression, a prudent antibiotic administration is highly recommended (https://ec.europa.eu/health/amr).

The economic burden of mastitis and its negative impact on milk quality (changes in chemical and organoleptic proprieties), are well-known by dairy producers and by all players in the value chain. In this view, attention has been given to accurate detection and prevention of mastitis (More, 2009), and a multitude of tests and technologies are currently available or in development, to detect the disease as soon as possible (Table 1).

**Table 1:** Summary of diagnostic methods for mastitis indicative markers and pathogens.

**Principle**	**Test**	**Description**	**Strengths**	**Weaknesses**	**Comments**
**MASTITIS INDICATIVE MARKERS**					
**Evaluation of somatic cells (SC)** Detection of epithelial and leucocyte cells released in milk. Most employable method to diagnose sub-clinical mastitis Diagnostic specificity compromised as SC can be elevated in several physiological conditions. Does not provide information regarding the causative pathogen.	Laboratorial microscopy	Direct observation of milk in a microscopy slide. Cells are stained and counted.	• Direct visual inspection of SC present in milk	• Time Consuming • Dedicated equipment • Trained personnel	Available at analytical laboratories
	DeLaval™ cell counter	Fluorescent dye (propidium iodide), is used to stain the cell nuclei.Imaging technique.	• Rapid (time to results ~1 min) • Portable. Suitable for on-farm testing • Bulk tank/individual animals	• Investment on equipment	Available commercially.
	Fossomatic™ cell counter	Fluorescent labeling of SC using ethidium bromide to stain nuclear DNA. Flow cytometry.	• Automated • High throughput	• Significant investment on equipment • Trained personnel • Dedicated equipment • Requirements for dedicated space	Available commercially.
	Qscout™	Differential staining of SC. Imaging.	• Rapid (results obtained within minutes) • Portable. Suitable for on-farm testing • Differential staining potentially increases diagnostic sensitivity and specificity	• Requires further validation • Investment on equipment	Available commercially.
	Septer™	Detects and counts SC by Coulter counter method	• Handheld device suitable for on-farm testing	• Requires further validation • Requires pre-sample treatment	Available commercially.
	California Mastitis Test (CMT)	Indirect indicator for estimating SC in milk. The test reagent (Bromocresol-purple in detergent) reacts with cell's DNA to form a gel. The gel viscosity is proportional to SC present in a milk sample.	• On-farm testing • Cost-effective • Simple to use • Rapid	• Difficult to interpret • Sensitivity dependent on the nature of the pathogen causing the infection	Available commercially.
	PortaSCC^TM^	Measures activity of the enzyme esterase present in leucocytes.	• Cost effective • On-farm testing • Rapid • Simple to use	• Lower diagnostic performance when compared to other SCC tests	Available commercially.
**Detection of enzymatic activity** Measures the activity of enzymes reflecting tissue destruction such as NAGase and Lactate Dehydrogenase (LDH).	UdderCheck™	Measures the activity of LDH by detecting color changes.	• On-farm testing • Rapid (2 min) • Does not provide information regarding the causative pathogen.	• Lower diagnostic performance when compared to other SCC tests	Available commercially. Some tests available at laboratorial facilities
**Electrical conductivity** Measurement of changes in ionic composition of milk due to tissue damage. Does not provide information regarding the causative pathogen.	Milk Checker	Abnormal conductivity.	• Rapid (measurements within seconds) • Portable configuration compatible with on-farm testing	• Low diagnostic sensitivity particularly in bulk tank measurements • Portable configuration less sensitive when compared to SCC tests	Available commercially. Some readers are compatible with automated, in-line measurements.
**pH meters** Measures increases in normal milk pH (normal milk: pH = 6.7). Does not provide information regarding the causative pathogen.	Various	Colorimetric assay using bromothymol blue electrodes to measure ion concentration.	• Cost-effective • Portable, on-farm testing	• Low diagnostic sensitivity	Available commercially. Some readers are compatible with automated, in-line measurements
**DETECTION OF MASTITIS PATHOGENS**					
**Bacterial cell culture** Use of culture media to identify mastitis causing pathogens.Results are expressed as CFU/mL of milk.	Various	Milk samples are inoculated on culture plates and incubated for a defined period after which colony forming units are formed. Visual inspection to classify the pathogens. Further biochemical tests can be performed.	• Cost effective • (~ 3€ for a triplate) • Accurate identification of the pathogens • Can provide information regarding antibiotic susceptibility	• Time consuming. Time to results: ~ 16-48 h • Requires trained personnel • Requires special equipment and sterility conditions • Majority of tests performed at laboratorial facilities • Requires sample shipment for laboratorial analysis.	Available commercially. Some tests available for on-farm analysis but suffer from poor adoption from farmers.
**Molecular diagnostics** Identification of mastitis pathogens by detecting specific nucleic acid sequences (pathogen molecular signatures).	Various (e.g., PathoProof™)	*Amplification techniques:* PCR reaction. Fluorescence enables the real-time detection of the amplified products and is directly proportional to pathogen concentration in the original sample	• Enhanced sensitivity and specificity • Results obtained within 4 h • Relatively expensive (~ 20€/test in commercial systems which include reporting) • Can detect multiple pathogens	• Dedicated equipment • Performed in laboratorial settings • Trained personnel • Sensitive to several milk components. Requires sample pre-treatment to extract bacterial nucleic acids • When DNA is used, unable to differentiate between live/dead pathogens.	Available commercially.
	Various	*Sequencing Techniques:* Direct analysis (“reading”) of nucleic acid sequences. Unlike amplification, the all nucleic acid region that was amplified is known after sequencing.	• Enhanced specificity • Cost effective (~4€/sequence run), without result analysis	• Performed in laboratorial settings • Dedicated equipment • Trained personnel • Requires sample pre-treatment • Time to results can take several days • Very sensitive to the quality of the sample	Applied mainly to research purposes. Efforts are being made to develop portable and more user- friendly sequencers.
**BIOSENSORS AND LAB-ON-CHIP DEVICES**					
*Biosensors* Analytical devices that use recognition elements to detect biological molecules in a sample. The recognition event originates a measurable signal that can be detected by a transducer (optical, magnetic piezo or mass based). Potential for high sensitivity, specificity and shorter time to results.	Pemberton et al., 2001	Electrochemical assay using screen printed carbon electrodes containing NAGase and correspondent substrates.	• Reaction completed within 100 sec	• Not tested with real samples • Laboratorial settings	Research purposes. Buffer pH was adjusted to be compatible to biologic fluids
	Welbeck et al., 2011	SPR sensor (CM5 from Biacore). Competitive immunoassay. Pasteurized milk samples spiked with known concentrations of NAGase enzyme.	• No labeling requirements • The chip was re-usable • Reproducibility • Detection limit: 10 U/mL	• Not tested with real samples • Laboratorial settings	Research purposes. Requires further development.
	Akerstedt et al., 2006	SPR sensor. Affinity sensor to detect interactions between Hp and hemoglobin. Competitive assay. Tested with milk samples.	• No labeling requirements. • Detection limit:1.1 mg/L of milk. • Ability to detect Hp in 24/28 samples with weak to strong CMT reactions	• Sensitivity to small blood impurities present in milk • Requires samples treatment (elimination of fat) • Laboratorial settings	Research purposes. Requires further development.
	(Tan et al., 2012)	Electrochemical sensor. Immunoassay, with immobilized antibodies anti-Hp. Tested with ilk samples.	• Portable • No labeling requirements • Detection limit: 0.63 mg/L • Positive detection in 20 samples with sub-clinical mastitis (SCC > 5 × 10^6^ cells/mL) • Time to results: ~30 min	• Requires samples treatment (elimination of fat)	Research purposes. Requires further development.
	Peedel and Rinken, 2014	BIA system. Immunoassay performed in microcolumns, selective for *S. aureus*Fluorescence detection.Tested with mastitis milk samples.	• Detection of mastitis pathogens • Time to results: 17 min • Detection limit: 200 cells/mL	• Requires sample treatment (elimination of fat) • Detected non-viable cells	Research purposes. Requires further development.
	Lee et al., 2008	Microarray analysis for seven mastitis pathogensMolecular test based on DNA hybridization	• Multiplex detection • Detection limit in the range of 10^3^-10^5^ CFU/mL but dependent on the pathogen	• Requires extensive laboratorial procedures to extract and amplify bacterial DNA	Expected cost of the biochip was estimated between $15 and $20.
**Lab-on-chip devices** Integrated, analytical systems, combining biosensors or other detection structure (e.g., paper test strips), able to perform the different laboratorial operations in a single device.	Garcia-Cordero et al., 2010	Sedimentation microfluidic (rotational disc), exploiting the differences between fat and cell fraction in milk. Detection was in in the range of 5 × 10^4^-5 × 10^6^ SC/mL	• Automated, and portable, suitable for on-farm testing • Can measure 12 samples simultaneously • No requirements for sample pre-treatment or addition of reagents	• Time to results: ~15 min. • Indirect SC counts, based on the cell pellet volume.	A portable prototype has been developed
	Grenvall et al., 2012	Acoustophoresis in a microfluidic chip to separate SC from fat. Inspection of SC performed with phase-contrast microscopy. Detection was in in the range of 1–5 × 10^6^ SC/mL	• No requirements for sample pre-treatment or addition of reagents • Miniaturized sample treatment unit to separate cells from fat.	• Requires microscopy equipment to analyze the cells.	Once fat globules are separated, accuracy of the analysis was comparable to standard Fossomatic and Coulter counting methods.
	Kim et al., 2017	Microfluidic system containing dye reagents to stain SC, combined with a portable fluorescent microcopy to analyze the cells. The staining protocol is automated and assured by capillary-driven fluid flow. Detection limit in the range of 5.9 × 10^4^-1.2 × 10^6^ SC/mL.	• Automated and portable format • Time to results: 10 min	• Not tested with real samples. • Spiked milk samples with a leucocyte cell line	
	Duarte et al., 2016	Magnetic flow cytometry, combining magnetic beads conjugated with bacterial-specific antibodies, microfluidics and MR sensors. Immunological recognition. Real mastitis samples.	• Detection of two different milk pathogens: *S. agalactiae* and *S. uberis* • Miniaturized format • Detection limit: 100 CFU/mL	• Requires sample pre-treatment (elimination of fat). • Semi-quantitative (yes/no response)	Research purposes. Although the chip is miniaturized, the signal acquisition system requires bulky equipment.
	Choi et al., 2016	LoC device combining 3D paper-based microfluidics. Miniaturized heating elements for LAMP reaction. Colorimetric detection.	• Detection of pathogens (*E. coli*) • All the reagents stored on paper. • Steps for DNA extraction and amplification performed on the 3D paper microfluidics • Reaction time: 1 h • Detection limit: 10 cells/mL	• Not tested with real samples. • Spiked UHT milk	A portable prototype has been developed
	Dimov et al., 2008	PDMS based microfluidic for automated fluidic handling combining RNA extraction and amplification by NASBA. Fluorescence detection	• Detection of pathogens (*E. coli*) • Automated nucleic acid purification and amplification • Reaction time: ~30 min • Detection limit: 1,000/mL cells	• Not tested with real samples (*E. coli* lysates) • Requires external sample lysis • Requires external equipment for fluorescence acquisition	Research purposes

National programs have been established to monitor overall milk quality, to assure that milk meets the quality standards and to provide information for educated decisions regarding prevention and long-term management Automated milking systems available in most modern farms are usually equipped with sensors that can monitor altered milk proprieties (e.g., conductivity, color), hence they have the potential for an automated diagnosis of mastitis episodes. While improvements in performance are still required, the majority of the described systems (Hogeveen et al., 2010b; Steeneveld et al., 2015), do not provide information for potential causative pathogens. To expedite diagnosis, prompt treatment, and improve herd management, there is an increased demand for on-farm tests that could evaluate if a mastitis case is of infectious origin and identify the pathogen (Adkins and Middleton, 2018). In this view, recent developments on biosensors and automated biological techniques may play an increasing role for on-farm microbiological tests.

## Conventional Approaches for Mastitis Diagnosis

### Mastitis Indicative Markers

Common strategies for mastitis diagnosis depend on the disease status, whether mastitis is clinical or subclinical. In clinical mastitis, manifestations of the infection are present, thus well-trained and vigilant staff is crucial to help initiate treatment and control the severity and impact of the disease (Ashraf and Imran, 2018). On the other hand, the absence of clinical signs in subclinical mastitis makes it harder to diagnose. Indeed, in most herds, the incidence of subclinical mastitis is 15–40 times higher than the clinically visible forms (Seegers et al., 2003; Adkins and Middleton, 2018; Ashraf and Imran, 2018). Nevertheless, the persistence of pathogen agents in the mammary gland evokes an immunological response. Hence, several approaches based on the detection of the different immunological effectors/modulators as well as modifications in the chemical properties of milk, have been developed for the detection of subclinical mastitis (Viguier et al., 2009; Adkins and Middleton, 2018; Ashraf and Imran, 2018). In the presence of an infectious agent, leucocytes and epithelial cells produce chemoatractants, cytokines (e.g., IL-8, IL-1, TNF-α) and acute phase proteins (e.g., haptoglobin [Hp], serum amyloid A [SAA]), that attract neutrophils to the site of the infection. The latter, act by engulfing the invaders and destroying them by oxygen and protease dependent mechanisms, which results in the release of enzymes such as N-acetyl-β-D-glucosaminidase (NAGase) and lactate dehydrogenase (LDH). Being non-specific, these mechanisms also destroy some epithelial and leucocyte cells that are secreted into the milk, increasing the somatic cell count (SCC). Milk production decreases, and changes on milk pH, conductivity and water content may also occur (Viguier et al., 2009).

Monitoring of SCC concentration in milk is the most implemented indicator to monitor mastitis, especially, in subclinical forms (Addis et al., 2016). In general, SCC values above 200,000 cells/mL of milk are considered an indication of inflammation and subclinical mastitis. For farm milk commercialization purposes, most European countries established the limit of 400,000 cells/mL, whereas in the USA, the limit is 750,000 cells/mL, with a decrease in milk price as the SCC number approaches the legal limit. Above the limit, the milk is worth a lot less. The SCC level is therefore an important parameter assessed by dairy farmers and dairy associations (Schukken et al., 2003; Lam et al., 2009). The SCC concentration can be determined at laboratorial level by microscopy using cell staining protocols. However, these methods are time consuming, require high-quality equipment and skilled personnel (Viguier et al., 2009; Adkins and Middleton, 2018). Alternatively, cell counters are available, either based on imaging techniques, Coulter counting or flow cytometry. For example, the DeLaval^TM^ cell counter can be used in bulk tanks and/or individual animals. Here, the cells are stained with a DNA fluorescent dye and an image is captured to quantify the number of stained nuclei (www.delaval.com). Another example is the Fossomatic™ counter. The principle is based on flow cytometry, where fluorescent labeled cells flow within a sheath fluid into a flow cell. Cells are then eradiated with light at a specific wavelength and the emission photons, captured by a detector. The most modern equipment can measure 600 samples/h and is compatible with automated milking systems (www.fossanalytics.com). The Coulter principle is based on changes in electrical conductance of a cell suspension in an electrolyte, while passing into an aperture between electrodes. The system is sensible to the number and size of the flowing cells (Norberg et al., 2004).

Portable configurations for SCC analysis are available in the market. For example, the Septer™ cell counter from Merck is a handheld, affordable device that measures SCC with comparable results to flow cytometry analysis (www.merckmillipore.com). However, milk is a water/fat emulsion with proteins, carbohydrates and minerals as colloids, and other particles in suspension that can interfere with the analysis (Fox et al., 2015). Thus, most of the described methods require a sample pre-treatment (e.g., elimination of the fat globules, filtration), that hamper their widespread implementation near-cow side. At the farm level, a well-known, affordable, easy, and fast method to estimate SCC is the California Mastitis Test (CMT). Its principle relies on the addition of a reagent with sodium lauryl sulfate (detergent), to disrupt cell membranes and promote lysis. In contact with the released DNA, the reagent jellifies, forming a gel which is visible to the naked eye. Although with wide implementation, the test fails to provide quantitative results and is prone to false positives due to subjective interpretation. On the other hand, the test can be used as therapy follow-on in recovering animals (Lam et al., 2009; Adkins and Middleton, 2018). Another cow-side available test is the PortaSCC^TM^ from PortaCheck (www.portacheck.com). It uses convenient paper-based test strips to monitor the activity of the enzyme esterase, which is present in white blood cells. The latter are trapped in the test pads; a dye substrate in the same region of the pad is catalyzed by the enzyme, originating a blue color with intensity proportional to the concentration of cells in the sample. Although cost effective, the test showed poor sensitivity for low SCCs (Lam et al., 2009; Viguier et al., 2009). Recently, Advance Animal Diagnostics (AAD), introduced a differential SCC analyzer suitable to operate near cow-side. The QScout MLD test from AAD can differentiate between the different leucocytes (lymphocytes, neutrophils, and macrophages). Understanding the ratio between the different leucocytes (e.g., elevated number of neutrophils), can improve the specificity of mastitis diagnosis, especially in cases of subclinical mastitis (www.qscoutlab.com).

Udder infections can also be detected by analyzing other biomarkers, such as released enzymes reflecting tissue destruction. Colorimetric and fluorometric assays have been developed to detect NAGase or LDH activity; Hovinen et al. described a fluorometric assay based on the catalytic activity of the enzyme using the substrate 4-MUAG. NAGase releases 4-MU that fluoresces in acidic conditions (Hovinen et al., 2016). Hiss et al. reported a portable spectrophotometer to measure LDH activity in raw milk at the farm level (Hiss et al., 2007). The UdderCheck™ from PortaCheck measures the LDH activity using paper-based test strips and by monitoring color changes in the presence of an LDH specific substrate. Results are qualitatively compared with a color chart to assess the severity of infection (www.portacheck.com). Other potential biomarkers are currently under investigation, including acute phase proteins such as Hp, SAA (Pyörälä et al., 2011), and cathelicidins (Addis et al., 2016).

The concentration of sodium and chloride ions is increased in milk from infected animals due to the damaged epithelial cells and weakened milk/blood barrier. Additionally, potassium levels decrease, with all these changes leading to modifications in electroconductivity (EC), of milk and increased pH levels. These parameters are widely used to identify abnormal milk proprieties, potential mastitis cases and infer on general herd status. Dedicated EC meters can easily be incorporated in automated milking units and robots. Nevertheless, EC values can vary significantly between different animals, compromising the definition of thresholds for both healthy and non-healthy conditions (Norberg et al., 2004; Lam et al., 2009; Viguier et al., 2009).

Diagnostic sensitivity and specificity for the described methods have been thoroughly analyzed in several research reports (Djabri et al., 2002; Ruegg and Pantoja, 2013; Corti et al., 2017). For example, diagnostic specificity of SCC analysis was found to be often compromised by false positives. The SCC parameter can be influenced by many factors, including animal stress, nutrition, stage of lactation, parity, and the quality of the fraction of milk sampled. Overall, the most accurate relationship between subclinical mastitis and SCC exists at the quarter level with sensitivities between 30 and 89% and specificities between 60 and 90%. CMT, when properly interpreted, could provide early diagnosis with high accuracy. However, both tests depend, at a certain degree, of the infectious agent. Generally, SCC provides enhanced diagnostic performance when compared to LDH and NAGase activity and, at the quarter level, EC meters perform poorly when compared to CMT or SCC counts (Corti et al., 2017; Adkins and Middleton, 2018).

### Detection of Mastitis Pathogens

Although the above-mentioned tests are indicative of disease, they fail in specifying the causative pathogen and hence, cannot support an educated treatment decision. The latter is of critical importance to control antibiotic administration and to initiate better management strategies such as avoiding spreading of contagious agents.

About 90% of pathogens responsible for udder infections are environmental pathogens, commonly present in the environment. Bacteria are amongst the most representative group of mastitis pathogens with *Staphylococcus aureus, Escherichia coli, Klebsiella* sp. and *Streptococcus* sp. causing the greatest losses of milk. The group of contagious agents comprises *S. aureus, Streptococcus agalactiae, Streptococcus dysgalactiae, Streptococcus uberis, and Mycoplasma* sp. Other common pathogens include *Corynebacterium* sp., coagulase-negative staphylococci and *Pseudomonas aeruginosa*.

Fungi are a less frequent cause of mastitis, with fewer reported episodes, most often found in farms with poor environmental and hygienic conditions. Similarly, contamination with micro algae belonging to the genus *Prototheca* are described, usually associated with poor milking conditions and prolonged antibiotic therapy (Zadoks et al., 2011; Klaas and Zadoks, 2018).

As stated earlier, the success of mastitis treatment is dependent upon the pathogen associated with mastitis. For example, whereas intramammary antibiotic therapy improves the rate of cure in cows infected with coagulase-negative staphylococci, and environmental streptococci, antibiotic's use is not recommended for cows with *E. coli* associated mild and moderate clinical mastitis (Ganda et al., 2016). In this view, the correct identification of the causing pathogen is critical for a targeted therapy.

#### Culture Methods

The gold-standard for the identification of mastitis pathogens are culture-based techniques. Results rely on incubating a known volume of milk in culture plates, for at least 18 h at defined temperatures to promote growth. Once finishing the growth period, colony forming units (CFU) are counted, and an analysis of the colony phenotype is performed to identify the agent. When necessary, additional biochemical tests can also be made. Most pathogens readily grow on a variety of available culture media, either aerobically (great majority) or anaerobically (e.g., *Mycoplasma* sp.). Culture plates are commercially available and relatively inexpensive. Specific culture media can be used to promote growth of specific microorganisms. Pathogen identification can be accomplished using milk from bulk tank or at the cow/quarter level. At the bulk tank level, preliminary CFU counts should be <5,000–10,000 CFU/mL with penalties in price as bacterial counts increases up to 20,000 CFU/mL (Murphy et al., 2016). At the cow/quarter level, the general recommendation is that starting from 0.01 mL of milk, bacterial counts should be <100 CFU/mL (Adkins and Middleton, 2018).

The major drawbacks associated with bacterial culture are related with sterility demands in order to prevent growth of mastitis non-related pathogens, requirements for specialized equipment and the need for skilled operators to correctly execute the microbiology techniques and interpret the phenotypic results. Additionally, the methodology often requires prolonged growth periods (up to 48 h), and is prone to false negatives, with reported probabilities for a false-negative result of 20–50% (Ashraf and Imran, 2018).

To expedite the methodology's use on a routine basis, on-farm culture kits have been developed. Initially established to distinguish between major group of pathogens (e.g., Gram-negative/Gram-positive), the more sophisticated ones can further differentiate between *Staphylococcus* sp. and *Streptococcus* sp., and specifically identify *S. aureus* (Ganda et al., 2016). Available tests usually comprise selective media with the aim of simplifying the interpretation of results from non-trained individuals. For example, the Accumast™ system uses three chromogenic media in a single plate (tri-plate), to distinguish between staphylococci, streptococci, and Gram-negative bacteria. The chromogens incorporated in the culture media are cleaved by specific bacterial enzymes, generating a visible change in color (Ganda et al., 2016; Lago and Godden, 2018). The Virbac test SpeedMam Color™ (chromogenic media), offers the possibility not only to detect different bacteria but also to analyze antibiotic sensitivity for fourteen antibiotics (www.bvt.virbac.com/).

While validation of such systems is currently ongoing, their diagnostic value is already recognized, particularly with respect to distinguishing between Gram-positive and Gram-negative bacteria, with sensitivities and specificities higher than 80% (Lago and Godden, 2018). Nevertheless, time to results is still high (16–24 h), and their adoption by farmers requires investment on dedicated facilities, equipment and personnel training (Adkins and Middleton, 2018).

#### Molecular Methods

The high frequency of false negatives using culture-based methods encouraged the development of molecular diagnostic tests which can provide high test sensitivity and specificity as well as detection of growth-inhibited and non-viable bacteria (Taponen et al., 2009; Duarte et al., 2015; Klaas and Zadoks, 2018). The use of polymerase chain reaction (PCR), to detect mastitis pathogens is known to be highly sensitive and specific, providing accurate pathogen identification, including those that do not grow using conventional culturing techniques. For example, in one study, Bexiga et al. reported bacteria detection by PCR in samples classified as negative by bacterial culture (Bexiga et al., 2011). Moreover, using PCR, results can be obtained within a few hours (Koskinen et al., 2009; Viguier et al., 2009; El-Sayed et al., 2017).

Since the beginning of the twenty-first century, several studies have reported the successful amplification and detection of mastitis pathogens using PCR based methods, targeting the 23S and 16S rRNA spacer region sequence (Phuektes et al., 2001; Riffon et al., 2001; Gillespie and Oliver, 2005). Riffon et al. described an assay to detect *E. coli, S. aureus, S. agalactiae, S. dysgalactiae, S. parauberis*, and *S. uberis* (Riffon et al., 2001), and Phuektes et al. presented a multiplex PCR assay for the simultaneous detection of *S. aureus, S. agalactiae, S. dysgalactiae*, and *S. uberis* (Phuektes et al., 2001). Later, Graber et al. used real-time quantitative PCR (qPCR), for detection of *S. aureus* by targeting the *nuc* gene (Graber et al., 2007), and a two-tube multiplex PCR assay for simultaneous detection of 10 bacterial species, *S. aureus, S. chromogenes, S. epidermidis, S. sciuri*, S*. haemolyticus, S. simulans, S. agalactiae, S. dysgalactiae, S. uberis*, and *E. coli* in milk, was reported by Shome et al. (2011).

Commercial assays for the detection of bacterial DNA in mastitic milk using PCR techniques have been used for more than one decade. One of the most used is the PathoProof™ Mastitis PCR Assay from Thermo fisher (www.thermofisher.com). This test is performed directly from raw or preserved milk samples and contains all the necessary reagents for the DNA extraction and amplification. It targets mastitis-causing pathogen's species or species groups and the β-lactamase penicillin resistance (*blaZ*) gene in *staphylococci* (including *S. aureus* and all major coagulase negative *staphylococci*) (Koskinen et al., 2009). Recently, Qiagen introduced a multiplex qPCR (Bactotype®) (www.quiagen.com), for the identification and differentiation of DNA from the three most contagious mastitis-causing pathogens *S. agalactiae, M. bovis, and S. aureus*. Koskinen et al. evaluated the PathoProof™ Mastitis assay using 1,000 milk samples from animals with either clinical or sub-clinical mastitis. In clinical samples (*n* = 780), bacterial culture was able to identify the udder pathogens in 77% of the samples whereas the qPCR PathoProof Mastitis kit detected the presence of bacteria in 89% of tested samples. In subclinical samples (*n* = 280), bacterial culture yielded positive results in 83% of samples while the qPCR kit yielded positive results in 91% of the analyzed samples (Koskinen et al., 2010).

Despite the short turnaround time for results, PCR is difficult to implement on-farm due to sterility requirements, the need for complex equipment and trained personnel. Additionally, the presence in milk of known PCR inhibitors (e.g., calcium, fat, high protein content), requires dedicated DNA extraction protocols to provide high quality results (Koskinen et al., 2009, 2010). For this reason, PCR-based techniques are most often performed in central laboratories.

Alternatively to standard PCR and qPCR techniques, loop-mediated isothermal amplification (LAMP), has been described as a great promise for rapid on-farm diagnostics (Cornelissen et al., 2016; Li et al., 2017; Klaas and Zadoks, 2018). This method is faster than PCR, less expensive, highly specific for the target sequence and less demanding in terms of the quality of the template and complex instrumentation. Ultimately, as an isothermal amplification technique, it could be implemented on field settings, requiring only a water bath or heat block for the reaction to occur (Mori and Notomi, 2009; Bosward et al., 2016; Lee, 2017). LAMP assays have been described for mastitis pathogens such as *S. aureus, S. agalactiae*, and *S. uberis* from bovine mastitis milk samples (Zhang et al., 2012; Zhao et al., 2013; Bosward et al., 2016; Cornelissen et al., 2016; Sheet et al., 2016).

Nowadays, the advances in next-generation sequencing (NGS) has opened a new window for the development of new genotyping methods to identify mastitis infectious agents, as NGS is becoming more available and affordable. In 2018, Anis et al. described a study using target-specific primers for PCR-mediated amplification with the NGS technology in which pathogen genomic regions of interest were enriched and selectively sequenced from clinical samples. This method allowed the successful detection of multiple bovine pathogens in clinical samples, including some additional pathogens missed by routine techniques because the specific tests needed for the particular organisms were not performed (Anis et al., 2018). This result demonstrates the feasibility of the approach and indicates that it is possible to incorporate NGS as a diagnostic tool in a cost-effective manner into a veterinary diagnostic laboratory and that likely in a near future NGS sequencing can be used as a tool in the routine identification of mastitis related microorganisms.

## Emerging Technologies

### Biosensors and Potential Applications in Mastitis Diagnosis

As discussed above, conventional methods for pathogen detection and identification are generally either time-consuming (culture and colony counting), or expensive (molecular methods), for the farmers in rural areas. Recent advances in micro- and nanotechnologies have led to the development of a new class of analytical systems—biosensors. Improved microfabrication techniques and novel nanomaterials with enhanced sensing capabilities or coupled to biomolecules, to work as reporters or signal amplification systems, are in the basis of more integrated biosensors for *in-situ* food analysis (Pérez-López and Merkoçi, 2011). The inclusion of nanostructures such as carbon materials (e.g., nanotubes, graphene sheet), metal nanoparticles (e.g., gold, silver, metal oxides) in different shapes (e.g., beads, rods, wires, disks), and many other structures, has demonstrated to improve transduction, aid in biorecognition and promote signal amplification.

**Figure 1:**
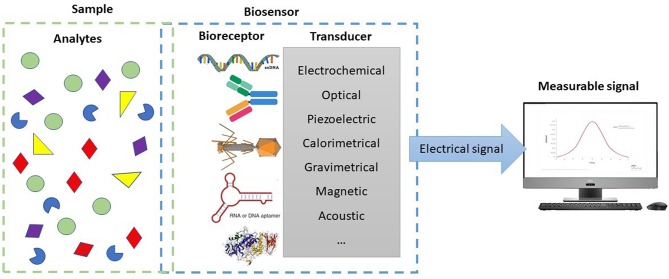
Schematic representation of the elements of a biosensor.

Biosensors are devices at the interface of biology with microsystems technology, which combine a biological element (bioreceptor) with a physical transducer, the sensor (Alhadrami, 2018). In general, when the biological recognition element interacts with a target molecule, the interaction creates a measurable signal that can be converted into data by the integrated transducer (Figure 1). There are several types of transducing principles, but the most commonly studied and in use for pathogen detection are electrochemical (Rotariu et al., 2016; Zhang et al., 2019), optical (Yoo and Lee, 2016), or fiber optic surface plasmon resonance (SPR) (Dudak and Boyaci, 2009) and piezoelectric (mass-based) (Pohanka, 2018). Calorimetric, gravimetrical, magnetic, and acoustic sensors are among other examples (Welbeck et al., 2011; Valderrama et al., 2016; Umesha and Manukumar, 2018). Depending on the type of transducer and nature of the target entity, different recognition elements (probes), can be used (Morales and Halpern, 2018). Single stranded oligonucleotides, antibodies or parts thereof, and enzymes are common examples of recognition elements used in sensing bacterial contamination. Less frequent, but emerging as promising alternatives, are bacteriophages, aptamers, peptide nucleic acids (PNAs), and other engineered affinity ligands, such as artificial binding proteins and molecularly imprinted polymers (MIPs) (Vidic et al., 2017).

In sum, a blend of these elements (transducer + bioreceptor + nanomaterial), has the potential to originate smaller, smarter, faster, and eventually cheaper technologies capable of performing complex molecular assays required for the diagnosis of microbial infections. Miniaturization would support their use either on a farm laboratory or as portable systems in the field. Automation and low hands-on time facilitate its use in a more generalized way by untrained farmers. Shorter time to results due to less sample preparation requirements and higher sensitivity leads to prompter treatment decision and improved approaches for herd management. Lower costs and reduced expenses in sample transportation, comparing to centralized lab analysis, make such technologies attractive to lower added value markets, with low-profit margin products, such as the veterinary market of food-producing animals and particularly the dairy industry.

Several review articles have been written covering the advances and trends in biosensors for the detection of pathogens and other contaminants (e.g., toxins, antibiotics), in food products of animal origin (Bahadir and Sezgintürk, 2017; Gaudin, 2017; Vanegas et al., 2017; Vidic et al., 2017; Abbasian et al., 2018; Neethirajan et al., 2018). Milk, mostly as a food-product, is referred as one of the target samples and is the basis of several proof-of-concept applications for many devices under development. However, for pathogen detection using milk as target sample and in particular, for bovine mastitis diagnosis, results are still limited (Mortari and Lorenzelli, 2014). Even so, the need to provide the milk supply chain with rapid, portable, and cost-effective biosensors for pathogen detection is a latent concern. In 2006 a consortium of a European project, the Pathomilk project (Grant agreement ID: 30392), joining dairy associations and other milk related stakeholders, got over 1.7 M€ in funding to develop a rapid, multi-pathogen analyzer for detection of the most common pathogens found in milk. The system comprised an innovative biosensor based on a DNA-hybridization method, using surface plasmon resonance as the detection technique. To the best of our knowledge no relevant outcome from the project was attained.

One of the reasons for the limited existence of fully automated, self-contained biosensing solutions for mastitis milk analysis is certainly related with the complexity of the milk matrix. Despite biosensors being characterized by direct analysis of complex samples with minimal or no sample preparation required, when it comes to milk, its heterogeneous composition (e.g., fat, proteins, carbohydrates, inorganic particles, somatic cells), is known to interfere or inhibit most analytical processes. Furthermore, mastitic milk matrix can be even more complex in terms of rheology and composition than milk from healthy animals. Elevated SCC associated with altered protein quality, change in fatty acid composition, lactose, ion, and mineral concentration, increased enzymatic activity, higher pH, and blood traces may negatively affect biological reactions (Ogola et al., 2007). Antibody-binding reactions in immune-based biosensors are affected by fat and other compounds in milk. Also, both DNA extraction techniques (Rossen et al., 1992) and PCR are well-known to suffer from interferences from milk matrix. In PCR, major inhibitors are described to be calcium ions (Bickley et al., 1996) and plasmin, which degrades the polymerases (Powell et al., 1994). Therefore, sample preparation, such as filtration of fat, somatic cells (SC) and excess proteins, would improve detection methods in general.

Besides bacterial cells and their nucleic acids, other biomarkers are considered in the diagnose of bovine mastitis. Enzyme b-N-acetylglucosaminidase (NAGase) and haptoglobin (Hp) are common targets in the development of biosensors. Back in 2001, Pemberton et al. presented an electrochemical assay for NAGase, using bare screen-printed carbon electrodes. The biosensor demonstrated the electrochemical determination of NAGase enzymatic activity at concentrations of 10 mU/mL in buffer solutions (Pemberton et al., 2001). Immunosensors were also reported to determine NAGase concentration. Welbeck et al. have used a single chain fragment variable antibody (scFv), against NAGase combined with a SPR platform and obtained a limit of detection of 10 U/L in spiked pasteurized milk samples (Welbeck et al., 2011). An SPR biosensor assay was developed by Akerstedt et al. to detect the interaction between Hp and a standard hemoglobin (Akerstedt et al., 2006). The precision of the assay was determined by analysis of bulk tank milk spiked with human Hp and the limit of detection determined to be 1.1 mg/L. Tan et al. have explored the same biomarker and method, using an amperometric biosensor and milk samples, spiked with bovine Hp (Tan et al., 2012). However, the smallest blood impurities in the milk may interfere with these assays by reducing the inhibitory effect of free Hp on the fixed amount of added hemoglobin, and samples containing blood cannot be analyzed with this method.

Regarding bacterial cells, due to their low concentration in small volumes of sample, high sensitivity can only be achieved through sample pre-enrichment, concentration, and/or amplification of bacterial DNA. Since, sample enrichment by cell culture methods is time consuming, normally taking place overnight, it does not respond to the short time frames required from on-site biosensors. In view of this, on-chip concentration is a more attractive approach.

Flow-based biosensors, besides the advantage of rapid results and easy system reusability, are frequently associated to an upstream target concentration step. Magnetic flow cytometers are good examples as discussed on the Lab-on-Chip section. Other type of flow-based optical immune-biosensing was reported by Peedel and Rinken who described the integration of a Bead Injection Analysis (BIA) in the detection system. *S. aureus* bacteria have been quantified in freshly spiked milk in about 17 min with a detection limit of 200 cells/mL (Peedel and Rinken, 2014).

Additionally, DNA amplification-based biosensing devices have been widely investigated (Fusco and Quero, 2014). PCR products after amplification may follow to DNA microarrays or other hybridization-based detection principles, which rely on the immobilization of single stranded DNA probes onto the sensor surface. The probe sequences can be robotically spotted over the sensing sites and when complimentary target amplicons specifically hybridize, a signal is generated in the transducer. Lee and coworkers developed a PCR-based DNA microarray for the multiplexed detection of seven known mastitis-causing pathogens within 6 h. The biochip consisted of 4 manually assisted steps: bench-top DNA macro-extraction of bacteria, DNA amplification by conventional off-chip PCR (in-tube), DNA hybridization, and colorimetric reaction detected with naked eye. The test samples were bacterial broth of reference strains, from which DNA was extracted using a commercial kit. The detection limit of the method was found to be in the range of 10^3^-10^5^ CFU/mL (Lee et al., 2008).

**Figure 2:**
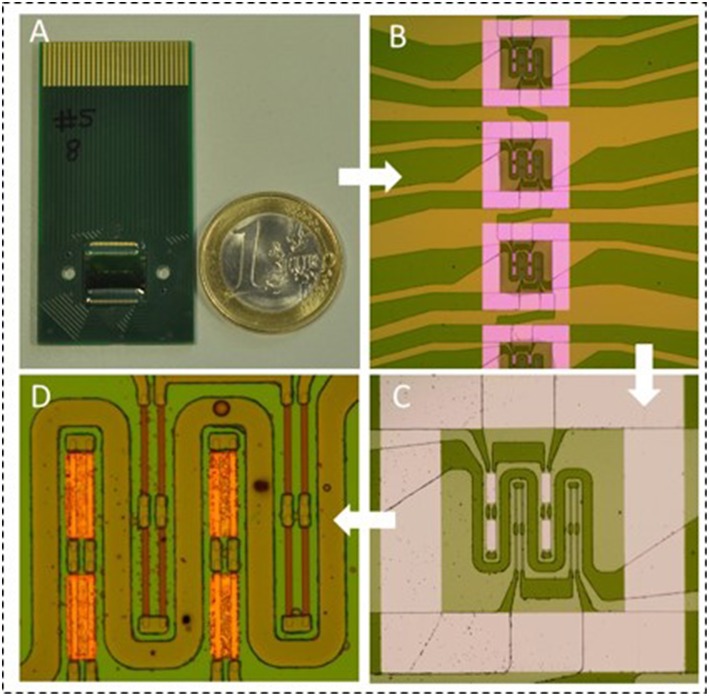
**(A)** Picture of the magnetoresistive biochip developed by Magnomics S.A. **(B)** Zoom-in of some of the 15 sensing sites for automatic spotting of biorecognition probes. **(C)** Detail of one sensing site with 2 sensors. One active sensor (gold coated) and one reference sensor (bare). **(D)** Zoom-in of the sensors. Each sensor is composed of 8 sensor segments. The sensors are surrounded by a current line for magnetic attraction of the magnetic particles.

A more recent type of biosensors, compatible with microarray formats and multiplexed analysis are the magnetoresistive (MR) sensors (Graham et al., 2004). MR sensors, such as spin-valves (SV), consist of a multilayer of magnetic and non-magnetic thin films, in which, the electrical resistance changes with an external magnetic field (Freitas et al., 2007). For labeling of the target molecules and transduction of the recognition events, superparamagnetic nanoparticles are used. In the presence of an external field these particles exhibit a magnetic fringe field detectable by the spin-valve in its proximity, causing a change in its electrical resistance. The sensor resistance variation is proportional to the number of magnetic particles bound to the captured target molecules, therefore allowing for a quantitative analysis. Reported MR biochips (Figure 2), may also integrate current lines as magnetic traps for assistance on magnetic particles' transport, concentration and detection (Martins et al., 2009). Such biosensors are also prone to an easy integration with microfluidics and electronics (Freitas et al., 2012) making them good candidates to the development of lab-on-chip (LoC) devices.

### Toward Lab-On-Chip Platforms

The combination of the miniaturized sensors described above with microfluidics, culminated on the emergence of a new era for diagnostics. The characteristic miniaturized fluidic network, of channels, chamber-integrated valves, pumps, and sensing units is boosting the development of portable laboratories, where all the necessary steps from sample-in to results can be performed, on-site, automatically, with minimum hands-on time. Such systems, usually referred to as lab-on-chip (LoC), hold the promise for lower assay costs, faster reaction times and identical diagnostic performance as their benchtop counterparts (Boyd-Moss et al., 2016). Dominant in the human diagnostic field, the veterinary, environmental, and food sectors have raised increased attention from the LoC-developer community due to regulatory and public pressures on increased quality standards and animal welfare, as well as the need to control infectious outbreaks (Neethirajan et al., 2011). In fact, the paper-based test strips previously described, represent one of the simplest concepts of LoC devices. However, taking advantage of microfabrication technology and integrated electronics, complex operations such as centrifugation, mixing, filtering, nucleic acid amplification, and labeling can be performed (Boyd-Moss et al., 2016).

**Figure 3:**
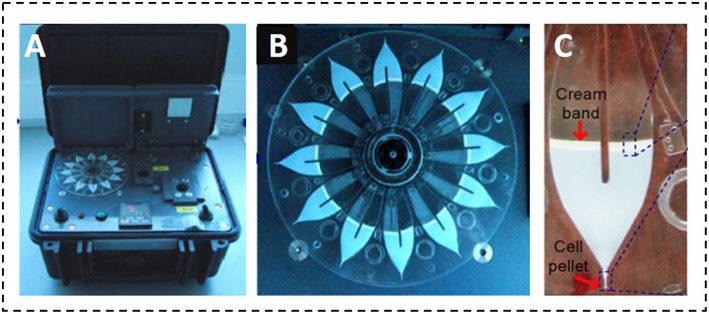
Portable microfluidic sedimentation cytometer proposed by Garcia-Cordero et al. **(A)** Portable reader to spin the disc; **(B)** Microfluidic CD cartridge showing the capacity for 12 milk samples; **(C)** Photographic capture of a centrifuged chamber showing the pellet and the cream band. Adapted with permission from Garcia-Cordero et al. (2010). Copyright 2010 Springer Switzerland AG.

In the context of mastitis diagnosis, a research report by Garcia-Cordero and co-workers described a portable microfluidic sedimentation cytometer for the analysis of SC. The approach was based on the centrifugation of milk samples into a closed-end microfluidic channel to form a visible cell pellet, proportional to the number of cells present in the sample. The system consisted of 12 independent channels, mounted on the footprint of a plastic compact disc (CD) (Figure 3). Time-to-results was 15 min, without sample pre-treatment steps, since cells could be separated from fat globules due to differences in density. Below 500,000 cells/mL the system showed a correlation of 0.92 when compared to a standard SC counter (Garcia-Cordero et al., 2010). Grenvall et al. used acoustophoresis in a 25 mm microchannel to effectively separate SC from lipid particles. The system required an external cell counter to count the cells. Nevertheless, the sample processing was simplified as no pre-labeling or centrifugation steps were required (Grenvall et al., 2012). Recently, Kim et al. combined a multi-functional chamber with a miniaturized fluorescence microscope. The system assured an automated sample delivery and a pre-stored, dried dye in the chamber enabled the *in-situ* staining of SC. Results showed a correlation factor of 0.9993 when compared to standard microscopy (Kim et al., 2017).

Combination of molecular techniques with microfluidics for pathogen detection is extensively described in the literature and was recently reviewed by Nasseri et al. (2018). Some platforms have even reached the market, with notable players commercializing products that are able to detect bacteria, fungi, parasites, viruses, toxins and other biomarkers, not only in body fluids (mainly blood, saliva, and urine), but also in food and environmental samples (Neethirajan et al., 2011; Volpatti and Yetisen, 2014; Nasseri et al., 2018).

**Figure 4:**
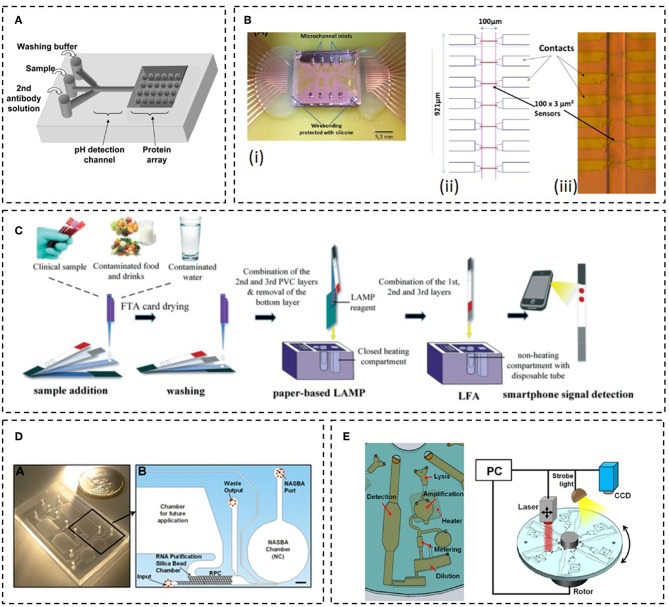
Examples of LoC systems for the detection of bacteria. **(A)** Schematics of the device proposed by Choi et al. Adapted from Choi et al. (2006); **(B)** Final device proposed by Duarte et al. with the MR chip bonded to the polydimethylsiloxane (PDMS) microchannels (i); Sensor layout showing the distribution of the Spin-valve sensors along the microchannels (ii) and a photograph showing a representative microchannel aligned over the sensors (iii). Adapted from Duarte et al. (2016); **(C)** 3D paper-based microfluidic proposed by Choi et al. Reprinted with permission from Choi et al. (2016). Copyright 2016 Royal Society of Chemistry; **(D)** Monolithic device proposed by Dimov et al. (inset **A**) and schematics showing the different unitary operations occurring inside the device (inset **B**). Adapted with permission from Dimov et al. (2008). Copyright 2008 Royal Society of Chemistry; **(E)** Scheme of the LoC device proposed by Kim et al. Adapted with permission from Kim et al. (2014). Copyright 2014 American Chemical Society.

Yet, reports regarding LoC detection of mastitis pathogens are lagging in the field. In one study, Choi and co-workers reported a LoC device to the simultaneous detection of microorganisms (*E. coli* 0157:H7 and *S. agalactiae*), antibiotics (dihydrostreptomycin and penicillin G), neutrophils, and pH in raw milk. The principle was based on immunological detection using a protein chip, coupled to a polydymethylsiloxane (PDMS) microchannel for sample and buffer insertion (Figure 4A). SNARF-1-dextran was used as pH indicator and immobilized on a sol-gel matrix along a region of the microchannel. The system required an external microscope and bacterial concentrations as high as 10^9^ cells/mL. Still, it was one of the first demonstrators showing the feasibility of LoC microfluidics in the analysis of milk bacteriology (Choi et al., 2006). Duarte et al. combined MR sensors with immunological detection to detect milk bacteria flowing inside a PDMS microchannel (Figure 4B). Magnetic nanoparticles (MNPs) were functionalized with antibodies in order to capture and distinguish *S. agalactiae* from *S. uberis*, directly from mastitic milk samples. The detection principle was based on the sensitivity of the MR sensors toward the magnetic fringe fields of the MNPs, while flowing above the sensors. When using a monoclonal antibody specific to *S. dysgalactiae* and comparing to a reference PCR method, the magnetic detection showed sensitivity and specificity values of 73 and 25%, respectively. The use of MNPs provided the additional advantage of bacterial concentration and hence, no pre-enrichment culture step was necessary to detect down to 100 CFU/mL (Duarte et al., 2016).

Integrated LoC systems for pathogen detection, combining sample treatment (using milk as target sample), nucleic acid amplification and detection are described in the literature. Although not directed toward mastitis diagnosis, the degree of integration and reported results deserve some attention as they could be fine-tuned, and potentially applied to detect mastitis pathogens at on-farm settings. For example, Choi et al. reported a 3D paper based microfluidic system, coupled to a handheld heating device for nucleic acid amplification (Figure 4C). In here, all the necessary reagents for DNA extraction and amplification by LAMP were stored on paper and all the reactions performed *in situ*. The device allowed for the detection of *E. coli* cells spiked in milk samples (from a grocery store, normal consumption milk), with a detection limit of 10 cells/mL, within a 1 h total reaction time (Choi et al., 2016). An approach based on centrifugational microfluidics, was pursued by Oh and coworkers. The steps for DNA purification and amplification (LAMP), were performed on the disc. Moreover, 5 amplification chambers provided the means for a multiplex detection. DNA extraction was performed using a commercial kit and the lysate introduced into the device to be purified by a bead-based method. The detection limit of the device was tested with *E. coli* O157:H7 cells, spiked in UHT milk samples and was calculated to be 2.7 × 10^4^ cells/mL (Oh et al., 2016). Dimov et al. incorporated solid-phase extraction and isothermal nucleic acid sequence-based amplification (NASBA), a transcription-based RNA amplification system, into a single cartridge (Figure 4D). The monolithic microfluidic platform that incorporates transfer-messenger RNA (tmRNA) purification, on-chip amplification (2 μL reactional volume), and real-time fluorescence detection was demonstrated in crude *E. coli* bacteria lysates containing 100 cells, within ~30 min from sample loading to result (Dimov et al., 2008). On-chip cell lysis was reported by Kim et al. using a rotational disc and paper test strips to detect the product of RPA amplification. Magnetic beads coated with specific antibodies were used for the simultaneously off-chip capture and concentration of the target cells (Figure 4E). However, in this work, *Salmonella enteritidis* spiked in 1 mL milk aliquots were lysed inside the device using a laser based assisted lysis. The reported detection limit in milk samples was 100 cells/mL (Kim et al., 2014).

**Figure 5:**
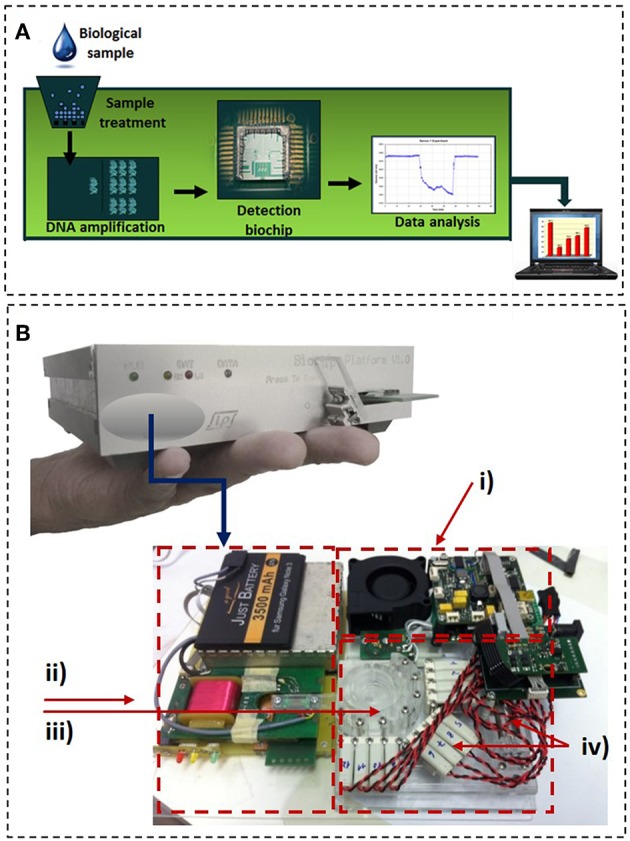
Magnomics S.A. device. **(A)** Schematics of the concept proposed by Magnomics S.A. **(B)** Prototype device developed by Magnomics S.A. highlighting the microfluidic units for sample treatment, nucleic acid amplification, and detection: (i) Thermocycling; (ii) Magnetic detection; (iii) Sample preparation unit; (iv) Integrated valves and pump.

As stated earlier, despite the number of research reports on LoC systems for bacterial detection using milk as the test matrix, to the best of our knowledge, few have addressed the problematic of on-farm pathogen detection in milking animals. Likewise, no commercial device is yet available on the market. Nevertheless, this window of opportunity is becoming attractive for some emergent start-ups. A handheld and easy to use device for the identification of different bacteria responsible for mastitis is under development by a Portuguese start-up (Magnomics S.A.). This LoC chip device is being designed to detect bacterial DNA and include all necessary laboratory steps (sample preparation, DNA amplification, and DNA detection), by combining microfluidics with MR sensors, inside a disposable cartridge. The cartridge can be calibrated to identify in a single shot several bacterial targets including, bacteria groups (Gram +/–), specific bacteria and antibiotic resistance genes. Such information will directly inform on the best treatment/antibiotic to prescribe within a maximum of 4 h. The test is also being designed to be easy to use for fast and flawless measurements, without the need of specialized personnel. In fact, the test will require only 3 operation steps: (i) insert the sample in the cartridge, (ii) insert the cartridge on the reader and (iii) press a button to start the measurement process. Three operations will automatically occur inside the disposable cartridge: (i) sample preparation, (ii) DNA amplification and labeling and, (iii) DNA detection using a magnetoresisitive-based micro-array (Martins et al., 2009). The sequence will be electronically controlled by the reader. In the last step the reader will also electronically acquire and analyze the signals (Germano et al., 2009), and finally, display the identification of the different parameters as a yes/no result (Figure 5A). The methods involve: (i) bacterial lysis, optimized in such a way that both Gram+ and Gram- bacteria can be lysed with similar efficiencies; (ii) DNA purification, accomplished by a solid phase extraction method, using magnetic nanoparticles as solid support. The magnetic particles can be easily captured and manipulated using magnets which facilitates the future integration of the sample preparation unit into a simple microfluidic cartridge. Furthermore, the developed chemistry avoided the usage of ethanol, commonly used in most of the DNA purification kits, due to its instability in small volumes, at room temperature, limiting the shelf life of the cartridge. Also, there are some commercial constraints of selling devices including ethanol; (iii) DNA amplification, performed using an end-point PCR with a specific set of primers targeting sequences of Gram +/– bacterial groups (Carroll et al., 2000), *S. uberis* (Shome et al., 2012), *S. aureus* (Brakstad et al., 1992) and the antibiotic resistance gene encoding for BlaZ (Olsen et al., 2006). These parameters were chosen as they provide relevant information for veterinarians on the best treatment in case of a mastitis diagnosis. Gram +/– helps deciding whether the treatment requires antibiotics (Lago and Godden, 2018). *S. uberis* and *S. aureus* are involved in the most infectious ailments requiring special care such as prolonged treatments, animal segregation, and careful disinfection to avoid contamination and the need for an eventual culling (Keane, 2019). The detection of BlaZ antibiotic resistance gene indicates that the bacteria involved in the infection may be resistant to β-lactam antibiotics and therefore other antibiotics should be used in treatment. The detection technology is based on the sensing of magnetically labeled amplicons by magnetic field sensors (Figure 5B), in particular, MR sensors (Freitas et al., 2012). The system is currently in development stage, with future actions focusing on the microfluidic integration of the sample preparation and amplification units before validation of the complete system.

## Conclusions and Future Trends

Mastitis or the inflammations of the mammary gland in dairy cattle has a tremendous impact on dairy industry economy, mainly due to milk losses or premature culling of the animals. As consequence of the milking activity by itself, it is extremely difficult to eliminate. While most cases are of bacterial origin, common measures rely on damage control, and administration of antimicrobials, mostly antibiotics. This fact causes additional stress over dairy industry stakeholders, as authorities and general public are demanding for a significant decrease on antibiotic usage. In this context, the existence of accurate diagnostic tools for constant surveillance and early detection of mastitis that could simultaneously identify the pathogens and direct treatment, represents an added value. The present article discusses the main approaches to tackle mastitis diagnosis. Some of the methods are commercially available while others are under research or development stage (main test features are summarized in Table 1).

In one side of the spectrum, a variety of diagnostic tools are available to detect altered milk proprieties at both physical, biochemical, and cellular level. An effort has been made to deliver these tools near cow-side, and in this way, accelerate diagnosis. In most modern farms, sensing tools were incorporated in automated milking systems and robots, capable of providing on-line measurements and, particularly in developed countries, national authorities, and local dairy associations have developed diagnostic plans comprising routine analysis, data extraction, and reporting to assure that milk is delivered with proper quality characteristics. However, most of these methods fail to provide information regarding the nature of the pathogens, thus the treatment plans based on antibiotics hardly changed over the past years.

Developments on microbiology sciences have enabled the widespread implementation of bacterial culture techniques and antibiotic sensitivity tests in most laboratories and some efforts were made to translate these techniques to on-farm settings, with some tests available on the market. Still, requirements for dedicated skills and equipment has hampered implementation. For pathogen diagnosis, the common practice still requires sample shipment to dedicated laboratories, increasing time to results. Molecular methods are well-established in laboratorial settings and hold the promise for faster and more sensitive results, with commercial tests like PathoProof™ being increasingly applied and validated in many farms. Still, when compared to culture methods, they remain expensive for an industry that fights, on a daily basis, with oscillations in milk prices. Moreover, the sample logistics from farm to laboratory remains.

Recent advances in biosensors and miniaturization techniques toward LoC devices can at least theoretically, provide microbiological, and molecular data on a faster manner, closer to the point of interest, at a lower cost. However, to the best of our knowledge, no LoC device targeting the detection of on-farm mastitis diagnosis has been demonstrated. Most of the reported systems still suffer from low to medium maturity levels which limits technological and performance capabilities, as well as price. Regarding technological capabilities, a clear need exists for integrated protocols, aiming at sample preparation, capable of dealing with the complexity of the real-world samples, and in this way, offer a competitive advantage over laboratorial techniques. As stressed in the present article, milk, is composed of many substances that can compromise the detection techniques either by culturing or by molecular methods, particularly fat. Additionally, the presence of clots and precipitates can seriously compromise any automated, fluidic handling strategy. Finally, considering sometimes that even low bacterial concentration can evoke a mastitis episode, sensitivity of the techniques must sometimes be pushed to the limit. Hence, the presence of potential inhibitors can seriously compromise the assay.

It is also true that the sensitive aspects regarding milk samples are common in other biological samples. In this view, the research and the LoC developer's community are trying to provide interesting and successful solutions in an attempt to achieve efficient, on-chip sample preparation strategies. It seems reasonable to assume that as these evolve, validation studies will provide more data regarding the accuracy of the employed techniques. With all these limitations, the actual price of the existent and more mature LoC technology remains relatively high, with vendors preferentially targeting the human market where higher values per test are usually more accepted. Nevertheless, the veterinary market is in high demand for new technologies addressing the problematic of bacterial infection, animal welfare and antibiotic stewardship, where mastitis represents a critical challenge. New players are emerging in the market and there is a clear opportunity for new developments in LoC devices for veterinary applications.

## Author Contributions

SM has organized the structure and content of the paper with a contribution in writing for all sections. VM, FC, and JG have contributed in the writing process for all sections, particularly for the Biosensors and MR in the writing of the Molecular methods section. CD and RB have participated in the writing process of the introduction and mastitis figures. SC and PF are group leaders and supervisors of the magnetics group at INESC-MN in the origin of this scientific report.

### Conflict of Interest Statement

SM, VM, FC, JG, and MR were employed by Magnomics S.A. The remaining authors declare that the research was conducted in the absence of any commercial or financial relationships that could be construed as a potential conflict of interest.
